# N-Acetyl-β-D-Glucosaminidase Analysis in Sheep Milk Can Detect Early Intramammary Infection with High Yields If Incorporated into Mathematical Algorithms

**DOI:** 10.3390/ani15030371

**Published:** 2025-01-28

**Authors:** Yolanda Miralles, Victoria Fornés, Amparo Roca, Raquel Muelas, José Ramón Díaz, Gema Romero

**Affiliations:** 1 Responsible Research Office, Miguel Hernández University (UMH), Avda. de la Universidad s/n, 03202 Elche, Spain; vfornes@umh.es; 2 Technical Support Service for Teaching and Research (SATDI), Miguel Hernández University (UMH), Avda. de la Universidad s/n, 03202 Elche, Spain; aroca@umh.es (A.R.); raquel.muelas@umh.es (R.M.); 3 Agro-Food and Agro-Environmental Research and Innovation Centre (CIAGRO), Miguel Hernández University (UMH), Ctra. de Beniel km 3.2, 03312 Orihuela, Spain; jr.diaz@umh.es (J.R.D.); gemaromero@umh.es (G.R.)

**Keywords:** NAGase, SCC, algorithm, sensitivity, specificity, AUC, accuracy

## Abstract

The aim of this study was to analyse the effect of intramammary infection (IMI) on N-acetyl-β-D-glucosaminidase (NAGase) enzyme values and to study its predictive capacity using different algorithms. To do this, 26 sheep free of intramammary infection were selected, and their somatic cell count (SCC), NAGase and bacteriological culture were monitored for 2 weeks. They were then subjected to a series of unhealthy situations (UHS) for the mammary gland, and these variables were monitored for a further 4 weeks. IMI was established in 12 of the 26 sheep. In the 4 days following infection, a very marked increase in enzyme values was observed in the infected glands. The algorithm that obtained the best result was the one that detected, in at least two of the first four days after infection, an increase in NAGase values greater than 30% compared to the average of the 3 days prior to infection, obtaining an area under the curve (AUC) of 0.90, an accuracy of 89.8%, a sensitivity of 95.2% and a specificity of 85.7%.

## Simple Summary

Mastitis is a major concern for dairy producers and society due to its economic and health implications, which is why it is necessary to develop methods for detecting the disease in its early stages. The aim of this study was to study the effect of the establishment of intramammary infection on the values of the enzyme N-acetyl-β-D-glucosaminidase, and to analyse whether, through the use of algorithms, this enzyme could be useful to solve this problem. On the one hand, it was detected that in the 4 days following infection, there was a very sharp increase in the values of this enzyme as a consequence of the infection. On the other hand, it was found that several of the algorithms studied highlighted the good predictive capacity of the enzyme since they allowed up to 89.8% of the observations to be correctly classified, ensuring a sensitivity of up to 95.2% and a specificity of up to 85.7%. Using this algorithm in farm-level biosensors, it would be possible to detect intramammary infections in the first 4 days of the disease, which could significantly reduce the costs associated with this pathology.

## 1. Introduction

Mastitis is a major problem affecting dairy sheep production. From an economic perspective, it can cause significant losses, as the disease causes a decrease in both the quantity and quality of milk, which also results in a decrease in cheese yield [1]. In addition, the cost associated with treating the disease and the possible need to replace the affected sheep increases the operating costs of the farm. In health terms, intramammary infections (IMI) represent a major problem due to the possibility of pathogenic bacteria or their toxins present in milk reaching humans through the consumption of contaminated dairy products [2]. Animal welfare is also compromised when cases of mastitis appear since the pain associated with inflammation of the mammary gland can cause stress and suffering in affected sheep, affecting their quality of life and their ability to engage in normal behaviours, such as feeding and resting [3]. Finally, mastitis also negatively affects the environment, since due to the decrease in milk production, there is lower efficiency in the use of natural resources and greater pollution of the environment by needing more animals to obtain the same output [4]. For all the reasons mentioned above, early diagnosis of mastitis in sheep is essential to reduce its negative impacts.

N-acetyl-β-D-glucosaminidase (NAGase) is an enzyme found mainly in the lysosomes of leukocytes and, to a lesser extent, in the epithelial cells of the mammary gland. This enzyme is released into milk as a consequence of cellular damage and the inflammatory response that occurs during mastitis; therefore, it could be considered a good indicator of the disease [5]. Other studies in cattle have also managed to discriminate between healthy glands and those with both subclinical and clinical mastitis [6,7]. It was also observed that NAGase values were higher in clinical mastitis than in subclinical mastitis [8]. In small ruminants, although to a lesser extent than in cattle, studies have also found that both NAGase and somatic cell count (SCC) values are higher in glands with mastitis [9,10].

NAGase levels are influenced by the type of pathogen causing mastitis, with higher values being found in glands infected by major pathogens [8]. In sheep, a difference was also observed in enzyme values depending on whether the glands were infected with streptococci or coagulase-negative Staphylococcus [11].

The aim of this study was to analyse the effect of the onset of intramammary infection in dairy sheep on the activity of the NAGase enzyme in milk, and to analyse different algorithms that include this enzyme, together with other variables, to determine which one could be the best for IMI detection.

## 2. Materials and Methods

### 2.1. Animal Management

This experiment was carried out in the small ruminant facilities of the Teaching and Experimental Farm of the Polytechnic School of Orihuela at Miguel Hernández University. This study included 26 Manchega sheep. The rearing system was intensive, and the animals were housed throughout the year in a 100 m^2^ corral (1.19 m^2^/animal) with access to an outdoor area during the day. At the start of the experiment, the average temperature was 24 °C, which increased to 27 °C on an average at the end of the experiment. The animals were fed daily throughout the trial with 2.5 kg of dry matter of complete ration (Unifeed), distributed in two doses per day, and had free access to straw and water. The diet was mainly composed of alfalfa hay, corn, soybean, barley and wheat. The reproductive rhythm followed was one birth per year; males were introduced in November with a ratio of one male to every 10 females. Birth took place in April, and the lambs were suckled until one month of age when they were weaned. During this month, the mothers were milked once a day to extract milk that was not consumed by the lambs. After this time, the glands were mechanically milked separately twice a day (8:00 a.m. and 4:00 p.m.) using a milking machine manufactured by the company Gea Farm Technologies (Bönen, Alemania). The milking parameters used were a pulsation rate of 180 beats/min, a pulsation ratio of 50%, and a vacuum level of 36 KPa. These parameters are adequate for the Manchega breed used, having been used in previous studies carried out by the team [12,13].

The care of the animals used in this study and the procedures performed were carried out in accordance with Spanish Royal Decree RD 53/2013 and EU Directive 2010/63/EU on the protection of animals used for experimental research and other scientific purposes.

### 2.2. Experimental Design

#### 2.2.1. Sampling Methodology

**Figure 1:**
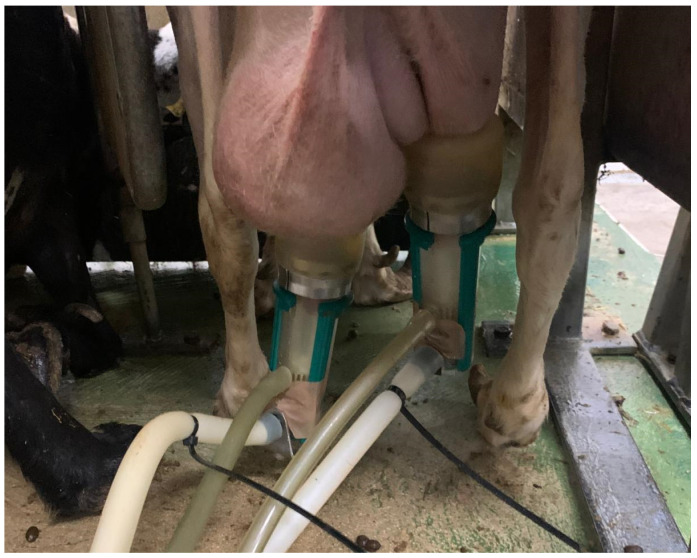
Photo of unhealthy situations.

The total duration of the experiment was 7 weeks, divided into two phases. The first period, called pre-experimental, started after weaning the lambs, lasted 1 week, and aimed to select healthy animals from the 50 lactating ewes available on the farm. The second period, called the experimental period, lasted 6 weeks and was divided into 2 stages. The first, lasting two weeks, aimed to determine the pre-conditions of the animals before the onset of infection. After these two weeks, the animals were subjected to a series of unhealthy situations (UHS) for the mammary gland that favoured the entry of pathogens through the teats at milking time in order to achieve controlled cases of IMI. Once these manipulations had been carried out, the second stage began, which lasted 4 weeks, to analyse the variations that occurred in the variables studied caused by the establishment of the infection. The unfavourable situations for mammary gland health that were practiced were as follows:

- Milking one healthy ewe after another with IMI after reducing the diameter of the short milk tubes (in this way, the volume of evacuation is reduced) and plugging the holes in the teat cups, which allows the introduction of an airflow that helps evacuate the milk (in this way, the immersion of the teat in the milk and the transmission of the infection are favoured) while causing cross-fluctuations allowing sudden air inflow through a teat cup (Figure 1)
- Raising the milking vacuum level to 40 kPa.
- Over-milking for 3 min.
- Not bathing the teats with iodine after milking.

**Figure 2:**
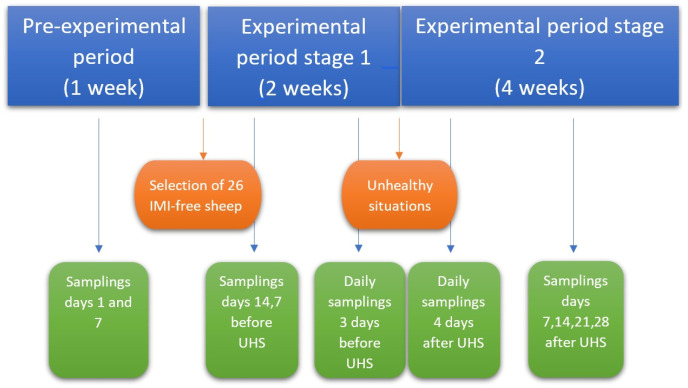
Timeline of experimental procedures. Abbreviations: IMI: Intramammary infection; UHS: unhealthy situation.

During the pre-experimental period, 2 samplings were carried out at an interval of 7 days in which the bacteriology, SCC and NAGase were analysed in the morning milking. After studying the results obtained, 26 IMI-free sheep were selected to participate in the experimental period. Three days after the last sampling in the pre-experimental phase, the experimental period began. During the first stage of this phase, sampling was carried out on days 14, 7 and 3 before the UHS, in which, as in the previous period, bacteriology, SCC and NAGase were analysed in the morning milking. From day 3 before the UHS until the day on which the manipulations were carried out, sampling was performed daily for these variables. In the second stage of the experimental period, after subjecting the animals to UHS for the mammary gland, daily bacteriological cultures were carried out until the presence of infection was confirmed and 6 more samples were collected on days 4, 7, 11, 14, 21 and 28 to check if the gland was still infected. SCC was also analysed daily for the next 14 days after UHS and on days 18, 21 and 28 after the establishment of infection. NAGase was also examined daily on the 4th day after UHS and on days 7, 14, 21 and 28 thereafter. A schematic of the chronology of experimental procedures is shown in Figure 2.

The sampling methodology was as follows: before milking in the morning session, after cleaning the teats with ethanol, a 5 mL aseptic sample was collected in a sterile tube for microbiological analysis. Each mammary gland was then milked separately. From this total milk sample, a 100 mL sample was extracted to analyse the enzymatic activity of NAGase and SCC.

#### 2.2.2. Variables Analysed

For bacteriological analysis, sheep blood agar plates were used, in which 20 µL of milk was sown and incubated at 37 °C. After 24, 48 and 72 h, the growth of the bacterial colonies was observed by analysing the density, growth rate, colony traits and appearance of the culture. Following the methodological guidelines proposed by the National Mastitis Council [14], cultures with five or more identical colonies per plate were considered positive. Cultures in which the growth of three or more different colonies was observed were classified as contaminated. In the samples that were positive, the microorganisms were isolated and frozen in milk with 2% glycerol at −4 °C. Identification of the bacterial genus was carried out at the end of the experiment, and Gram staining was performed. For Gram-positive colonies, the catalase test was performed. For staphylococci, the Apistah kit (BioMerieux, Marcy-l’Étoile, France) was used to identify the bacterial species, while for enterobacteria, the BBL Enterotube II kit (BD Diagnostic Systems, Heidelberg, Germany) was used. All the analyses were carried out by the same person.

SCC (1000 cells/mL) was analysed using the fluoro-opto-electronic method (Model Fossomatic 5000, Foss Electric, Hillerød, Denmark) from samples preserved in azidiol. These analyses were carried out at LICOVAL (Laboratorio Interprofesional Lechero de la Comunidad Valenciana). An interassay agreement of 5% was obtained, with analyses presenting a coefficient of variation of less than 4% (intra-assay) considered valid.

NAGase activity (µM/min per mL) was analysed following the method described by [15] using a fluorescence spectrophotometer (Fluostar Optima, BMG Labtech, Ortenberg, Germany). These analyses were also performed by the same operator. An interassay agreement of 15% was obtained, and these analyses were considered valid if the replicates had an intra-assay coefficient of variation of less than 15%.

#### 2.2.3. Definition of Gland Health Status

To classify the glands according to their health status, bacteriological analysis was used. Positive samples were considered infected, and negative samples were considered healthy. Clinical symptoms were not taken into account when defining the health status of the gland.

### 2.3. Statistical Analyses

Quantitative variable distributions were assessed using histograms and box plots. SCC (×1000 cells/mL) and NAGase (µM/min per mL) values were transformed using the natural logarithm to correct for the skewness of their distribution and ensure normality of the data when they were used in the regression models. However, in the case of descriptive statistics or non-parametric tests, the original scale variable was used to facilitate interpretation. Descriptive analyses were performed using the median (1st and 3rd quartile), while absolute and relative frequencies were used as qualitative variables.

The correlation between quantitative variables was assessed using the bootstrapping correlation coefficient in the R library rmcorr [16], given the non-independence of the observations. Confidence intervals were calculated using bootstrapping since they do not require distributional assumptions. The correlation between NAGase and SCC was calculated globally, considering the SCC levels (≤400,000 and >400,000 cells/mL), as well as for each of the time periods.

Univariate comparisons were made between GHS at different time points. *p*-values were adjusted using Holm correction for multiple comparisons. For inferential analysis, a mixed linear regression analysis was carried out considering log-transformed NAGase as a dependent variable. The following variables were considered in this study: days before or after infection (previous 14–7 days, previous 3 days, following 4 days, following 7–14 days, and following 21–28 days), the log-transformed SCC variable, and gland health status (GHS, with four levels. 0: Both collateral glands free of infection throughout the experiment. 1: Free of infection throughout the experiment whose collateral was infected during the experiment. 2: Infected during the experiment whose collateral remained free of infection. 3: Both collateral glands infected during the experiment.) and the first-order interaction between gland health status and time period with respect to the time of onset of infection was included. The random effect of glands (right or left) nested in the ewe was considered in the model to explain the clustering of mammary glands within the animals. An “autoregressive process of order 1” type correlation adjustment was used to model the variance structure between repeated measures from the same animal and gland. The final model provided the best fit for the data compared to other models that considered other covariances and hierarchical structures (evaluated using the Akaike information criterion).

To assess the predictive ability of NAGase values, logistic regression models were performed to predict IMI. The predictive ability of the models was evaluated using the Area Under Curve (AUC), the sensitivity, specificity, accuracy, positive predictive value and negative predictive value. Given that the onset of infection is expected to cause an increase in NAGase values, NAGase samples of each gland were classified as positive each day (from day 1 to 4 of follow-up after being exposed to UHS) according to different approaches that were proposed:

- Rule 1 (R1): The gland was classified as positive for each day when NAGase was higher than the reference mean value of the three previous days to the UHS by 5, 10, 20%, or 30% as follows:

$$\frac{NAGase_{\left\{D1,\;D2,\;D3,\;D4\right\}}}{RefMeanValue_{3d\;previous\;UHS\;}}>1.05;\;1.10;\;1.20;\;1.30$$

- Rule 2 (R2): The gland was classified as positive for each day when NAGase deviated from the reference mean value of the three previous days to the UHS by more than 2 or 3 times the standard deviation of the three previous days to UHS ($SD_{3d\;previous\;UHS}$), as follows:

$$NAGase_{\left\{D1,\;D2,\;D3,\;D4\right\}}-RefMeanValue_{3d\;previous\;UHS\;}>2\;or\;3\;SD_{3d\;previous\;UHS}$$

After classifying NAGase samples as positive or negative in each of the first four days of follow-up after being exposed to UHS according to these two approaches, three different strategies were considered:

- Strategy Any: Each gland was classified as positive when at least one of the four first days of follow-up overcame the corresponding threshold in each rule (R1 and R2); otherwise, the gland was considered negative.
- Strategy Some2: Each gland was classified as positive when at least two of the four first days of follow-up exceeded the corresponding threshold in each rule; otherwise, the gland was deemed negative.
- Strategy Some3: Each gland was classified as positive when at least three of the first four days of follow-up exceeded the corresponding threshold in each rule; otherwise, the gland was classified as negative.

These three strategies were proposed to assess the stability and consistency of NAGase values, as well as the proposed rules over four days of follow-up after the onset of infection. Analyses were carried out using nlme [17], rms [18], Calibration Curves [19], and R statistical software 4.4.0 [20]. A *p*-value < 0.05 was considered statistically significant.

## 3. Results

### 3.1. Mastitis Incidence

In the pre-experimental phase, 26 sheep free of intramammary infection were selected. Of these animals, 12 were infected after UHS, nine bilaterally and three unilaterally, with a total of 21 infected glands (Table 1). Therefore, an incidence of 42.3% at the gland level and 46.2% at the sheep level was obtained. Starting with healthy animals, the incidence throughout the experiment coincided with the prevalence after UHS. Regarding the pathogens causing the disease, the highest percentages of IMI were caused by enterobacteria (71.4%), including *Serratia marcescens* (9.5%), *Klebsiella pneumoniae* (19%), *Pantoea agglomerans* (9.5%) and others (33.4%). Some 28.6% of the infections were caused by staphylococci, with *S. xylosus* found in 9.5% of the glands, *S. caprae* in 4.8%, and 14.3% in other species.

### 3.2. Effect of the Onset of Intramammary Infection on the Study Variables

**Figure 3:**
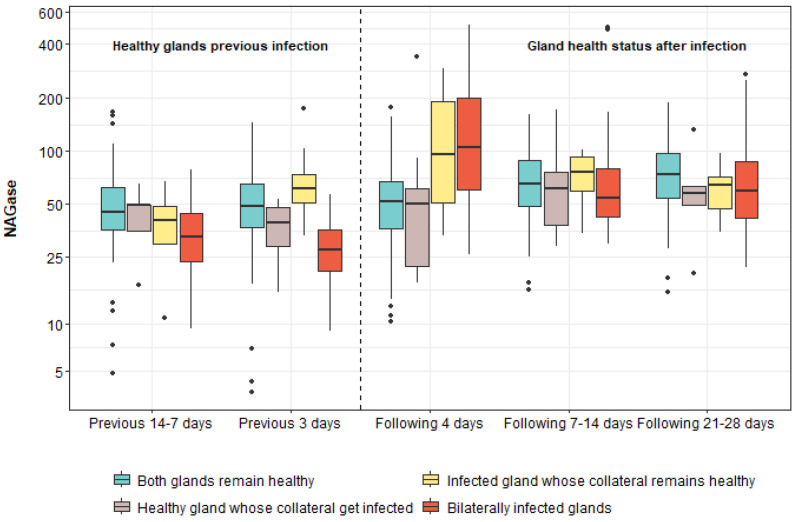
N-acetyl-β-D-glucosaminidase values (μM/min per mL) throughout the study, according to the health status of the sheep glands. Abbreviations: NAGase: N-acetyl-b-D-glucosaminidase. The black dots in the figure represent outliers in the data distribution.

Table 2 shows the effect that the onset of intramammary infection has on NAGase and SCC values. Regarding NAGase, in healthy glands (GHS = 0), a tendency towards a progressive increase in enzyme values over time was observed, and the same occurred in healthy glands whose collateral was infected (GHS = 1), although less markedly. The lowest values in each of the four groups according to the health status of the gland were observed on all days prior to performing the unhealthy situations for the mammary gland (27.44–61.46 μM/min per mL). In the SCC, the lowest values were also found on all previous days in each of the groups (44–97 cell/mL), except in the infected glands that presented the healthy collateral (GHS = 2), in which higher values were observed in the three previous days (367 cell/mL) than in the following 7–14 and 21–28 days (154.5 and 107.5 cell/mL). In both the glands infected with the healthy collateral and those infected bilaterally (GHS = 3), the increase in the values of NAGase and SCC occurred in a very marked manner in the 4 days following the onset of infection, at 95.59 μM/min per mL and 1,674,000 cells/mL for GHS = 2 and 105.69 μM/min per mL and 2,601,000 cells/mL for GHS = 3. These values decreased for both variables in the following two samplings, obtaining values in the following 7–14 days of 76.49 μM/min per mL and 154,500 cells/mL for GHS = 2 and 54.47 μM/min per mL and 85 500 cells/mL for GHS = 3. In the following 21–28 days, the values obtained were 64.84 μM/min per mL and 107,500 cells/mL for GHS = 2 and 59.85 μM/min per mL and 79,000 cells/mL for GHS = 3. These results are represented graphically for NAGase in Figure 3.

Multiple comparison analysis revealed significant differences in NAGase values three days prior to UHS between glands that developed bilateral infection and both healthy glands (*p* < 0.001) and infected glands whose collateral was healthy (*p* < 0.001). These differences were attributed to the lower values observed in bilaterally infected glands. On the other hand, four days after UHS, significantly higher NAGase levels were observed in unilaterally infected glands whose collateral was healthy compared to bilateral healthy glands (*p* = 0.019). Statistically significant differences were also detected between bilaterally infected glands and both healthy glands (*p* < 0.001) and healthy glands whose collateral was infected (*p* = 0.001), with bilaterally infected glands showing higher values. No statistically significant differences were observed between the other pairs of gland health states at different time points.

According to the mixed linear regression model that evaluated the effect of the covariates SCC, GHS and Time period (TP) on NAGase, a statistically significant positive relationship (0.28 CI95% [0.26, 0.31], *p* < 0.001) was observed with the log_SCC variable. A statistically significant interaction (*p* = 0.003) was also found between the variables GHS and TP; therefore, the establishment of IMI caused a greater increase in NAGase than in infection-free glands, as already observed in Table 2.

### 3.3. Correlation Between NAGase and SCC

Table 3 shows the correlation (r) between NAGase and SCC both globally and as a function of the SCC value. Analysing this with the total SCC values, a high positive correlation was found (r = 0.79), which decreased when analysed separately as a function of the SCC interval, obtaining higher values when the SCC was higher than 400,000 cells/mL (r = 0.65) than when it was lower than or equal to the SCC value (r = 0.41).

**Figure 4:**
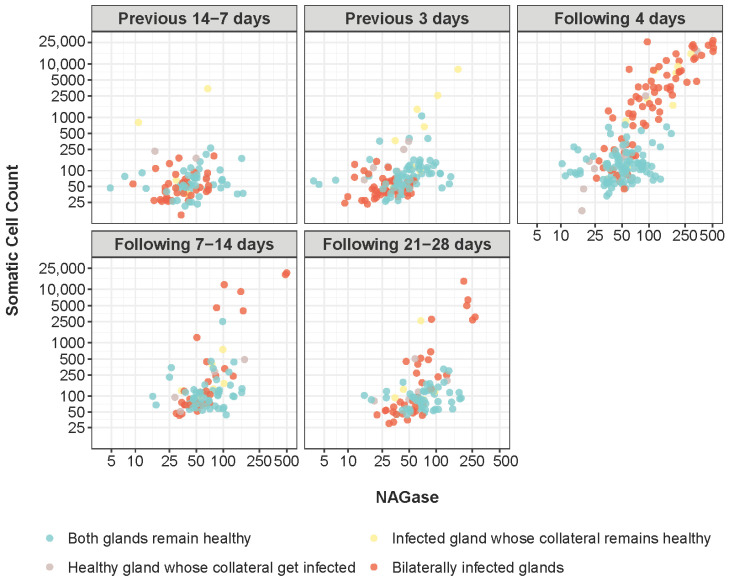
Correlation (r) between N-acetyl-β-D-glucosaminidase values (μM/min per mL) and Somatic Cell Count (×1000 cell/mL) in ewes’ glands according to health status and time period relative to the onset of infection.

Figure 4 shows the correlation coefficient between the two variables overall throughout the different periods related to the establishment of IMI. Although the correlation was positive in all cases, the highest values were found at 4 and 7–14 days after infection (r = 0.76 and r = 0.61). The lowest values were obtained 14–7 days prior and 21–28 days after infection (r = 0.31 and r = 0.33), followed by 3 days prior to infection (r = 55).

### 3.4. Predictive Models for Mastitis Detection

Table 4 shows all the predictive capacity indicators of the 18 proposed models (6 rules × 3 strategies), the area under the curve (which measures the predictive capacity of the model between 0 and 1), and accuracy (which is the percentage of observations correctly classified by the model):

- Strategy Any: The best indicators were offered by the Rule 1–30% model with an AUC of 0.79, which correctly classified 75.5% of the observations and ensured a sensitivity of 100%, but only a specificity of 57%.
- Some2 strategy: Higher AUC and Accuracy values were obtained, especially in the cases of Rule 1–20%, Rule 1–30%, Rule 2-2SD and Rule 2-3SD. Of these four models, there were two that stood out: Rule 1–30%, with an AUC of 0.90, which correctly classified 89.8% of the observations, ensuring a sensitivity of 95.2% and a specificity of 85.7%, and Rule 2-3SD, with an AUC of 0.86, which correctly classified 87.8% of the observations, ensuring a sensitivity of 85.7% and a specificity of 89.3%.
- Some3 strategy: Except for the Rule 1–5%, all the others offered good results. Rule 1–10% with an AUC of 0.85, correctly classified 83.7% of the observations, ensuring a sensitivity of 95.2% and a specificity of 75%; Rule 1–20% correctly classified 85.7% of the observations (AUC of 0.86), ensuring a sensitivity of 90.5% and a specificity of 82.1%; Rule 1–30%, with an AUC of 0.87, correctly classified 87.8% of the observations, ensuring a sensitivity of 81% and a specificity of 92.9%; Rule 2-2SD, with an AUC of 0.86, correctly classified 85.7% of the observations, ensuring a sensitivity of 85.7% and a specificity of 85.7%; and finally, Rule 2-3SD, with an AUC of 0.87, correctly classified 87.8% of the observations, ensuring a sensitivity of 81% and a specificity of 92.9%.

**Table 4:** Results for predictive models (AUC, Accuracy, Se, Sp, PPV and NPV) using both rules described in the Methodology section when applying algorithms to the NAGase measurements.

		RULE 1				RULE 2	
		5%	10%	20%	30%	2SD	3SD
Strategy Any	AUC	0.50	0.50	0.625	0.79	0.65	0.72
	Accuracy (%)	42.8	42.8	57.1	75.5	61.2	69.4
	Se (%)	100	100	100	100	95.2	90.5
	Sp (%)	0	0	25	57.1	35.7	53.6
	PPV (%)	42.8	42.8	50	63.6	52.6	59.4
	NPV (%)	-	-	100	100	90.9	88.02
Strategy Some2	AUC	0.60	0.67	0.80	0.90	0.79	0.86
	Accuracy (%)	55.1	63.3	77.6	89.8	77.6	87.8
	Se (%)	95.2	95.2	95.2	95.2	90.5	85.7
	Sp (%)	25	39.3	64.3	85.7	67.9	89.3
	PPV (%)	48.8	54.1	66.7	83.3	67.9	85.7
	NPV (%)	87.5	91.7	94.7	96	90.5	89.3
Strategy Some3	AUC	0.74	0.85	0.86	0.87	0.86	0.87
	Accuracy (%)	71.4	83.7	85.7	87.8	85.7	87.8
	Se (%)	95.2	95.2	90.5	81	85.7	81
	Sp (%)	53.4	75	82.1	92.9	85.7	92.9
	PPV (%)	60.6	74.1	79.2	89.5	81.8	89.5
	NPV (%)	93.8	95.5	92	86.7	88.9	86.7

Abbreviations: AUC = Area Under Curve; Se = Sensitivity; Sp = Specificity; PPV = Positive Predictive Value; NPV = Negative Predictive Value; Strategy Any: At least one of the first four days of follow-up overcomes the threshold; Strategy Some2: At least two of the first four days of follow-up overcome the threshold; Strategy Some3: At least three of the four first days of follow-up overcome the threshold.

**Figure 5:**
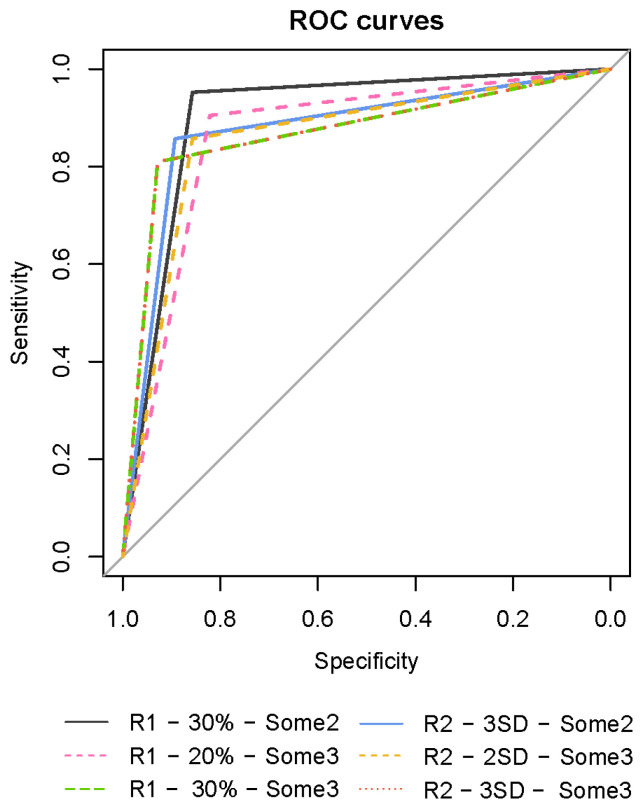
ROC curves of the best predictive models selected for detecting mastitis.

Figure 5 shows the ROC curves of the proposed algorithms for detecting mastitis. It can be seen that they are very similar, although in some cases, sensitivity is more important and in others, specificity.

## 4. Discussion

The incidence observed in this experiment (42.3% at gland level or 46.2% at sheep level) and the subsequent prevalence are higher than the average generally described in the literature, although these values may vary depending on the source consulted. We can find studies in which the infection occurred naturally, with very low values of 4.6% [21], average values of 19.3% [22], values closer to those obtained in this work of 39.9% [11], and even higher values (53%) [23]. The high prevalence in this study may be due to the fact that the infection was induced by unfavourable situations that were applied to the mammary gland, which increased the likelihood of the glands becoming infected. In studies carried out in goats with a similar experimental design, in which UHS was also carried out, an incidence of 50% for goats and 33.3% for glands was reported [24].

In this experiment, 71.4% of the glands were infected by enterobacteria and 28.6% were infected by staphylococci. Normally in the literature, in studies in which the infection occurred naturally, we find that enterobacteria infections occur in a very low percentage 3.4% [25], 3.8% [26], 0.9% [27] and that staphylococci are the bacteria that produce a higher percentage of IMI, 69.8% [25], 53.4% [26], 76.6% [27]. This difference may be due to the fact that it was not a natural infection, but rather that by causing unfavourable conditions in the mammary glands, the entry of bacteria that were in the environment surrounding the sheep (bedding, soil, manure) was facilitated, which could come into contact with the skin of the gland and subsequently colonise the interior.

Regarding the effect of the onset of intramammary infection on NAGase, it can be seen that, both in healthy glands (GHS = 0) and in healthy glands whose collateral was infected (GHS = 1), a tendency towards a progressive increase in the enzyme values was observed as the lactation stage progressed, as the first control was carried out a few days before 6 weeks after delivery and the last at 12 weeks. This trend corroborates a previous study by our research group in which it was observed that from week 6 after delivery, NAGase tended to rise physiologically until week 14 [28]. In the glands infected with the healthy collateral (GHS = 2) and in those infected bilaterally (GHS = 3), it was observed that, at 4 days after infection, there was a very sharp increase in NAGase values. This may have been due to the fact that, as a consequence of the acute inflammatory response caused by the infection, there was an increase in the release of the enzyme by leukocytes and damaged epithelial cells [5]. These results coincide with those of multiple studies in which it has been proven that infection increases NAGase values in both cows [5], goats [29] and sheep [30]. The values of the unilaterally infected glands increased by 50% compared to the previous 3 days, and almost four times more in those infected bilaterally. This suggests that bilateral infections can cause a more intense inflammatory response, possibly due to a stronger systemic response. Although the values decreased in the following two samplings, they continued to remain above the levels obtained in the previous days in the bilaterally infected glands. This sudden increase post-infection and stabilisation in later periods could have been due to two reasons. On the one hand, it is likely that the microbial load was low, and on the other hand, the animals were in a high immune state and were able to fight the infection quickly. These results coincide with those reported in another study, in which a prevalence of subclinical mastitis of 19% was observed at the onset of lactation, which decreased to 9.1% after 40 days, indicating that 93.8% of the sheep were cured spontaneously [31], and with [32,33] who observed a reduction in the prevalence with the progress of lactation.

The high positive correlation between SCC and NAGase when the values were analysed globally (r = 0.79) was very similar to that obtained in other studies, both in sheep (r = 0.85) [11] and cattle (r = 0.73) [5]. If we analyse the values separately based on the number of somatic cells, the correlation decreases, reaching the highest value when the SCC is higher than 400,000 cells/mL (r = 0.65). Although the correlation was positive throughout the study, the highest values were obtained at 4 and 7–14 days after infection (r = 0.76 and r = 0.61), which coincides with the moments at which the SCC reached the highest values.

Regarding the predictive value of the algorithms that included NAGase to detect intramammary infection, the results showed that, for this calculation, it was preferable to take only 4 days post-infection since these were the days when there was a more pronounced increase in the enzyme values. These values decreased in the following samples; therefore, if we took a wider range of time, the average would drop and the model would be less sensitive. When analysing the results of the predictive models, it was observed that Rule 1–5% generally did not work well, presenting a low specificity, since the NAGase values are quite dispersed and it is easy for them to deviate by more than 5% without the presence of IMI, so classifying as infected glands that present variations greater than only 5% compared to the baseline mean would lead to false positives. The best results were obtained for the Some2 strategy (R1-30% and R2-3SD) and the Some3 strategy (all except R1-5%), with AUC values greater than 0.85. To choose the best classification method, it should be considered whether a higher sensitivity or a higher specificity is desired. This will depend on the prevalence of IMI in the herd. If we have a low prevalence, it would be advisable to prioritise specificity more to avoid unnecessary treatments [34]. Although both the R1-30% and R2-3SD rules of the Some2 strategy and the R1-10%, R1-20%, R1-30%, R2-2SD, and R2-3SD rules of the Some3 strategy would give good results, the complete model at an overall level that obtained a higher AUC value (0.90), a higher sensitivity (95.2%), and a good specificity value (85.7%) is–R1-30% of the Some2 strategy. If we look for greater specificity, the R1-30% and R2-3SD models of the Some3 strategy are the ones with the highest values (92.9%), while maintaining good sensitivity values (81%). These values are higher than those obtained in studies in cattle, in which a sensitivity of 85% and a specificity of 84% were obtained for a cut-off value of 0.76 μmol 4 MU/min per μL of milk [5]. In sheep livestock, there are no studies on predictive models of NAGase, but the results obtained in this experiment are superior to those obtained in a previous study by our research group, in which non-infectious factors such as parity number, lactation status, milking and production were included in the algorithms, in which an AUC value of 0.80, a sensitivity between 64 and 73% and a specificity between 70 and 79% were obtained [28].

## 5. Conclusions

The results obtained in this study suggest that NAGase can be considered a good method for early detection of IMI since it can be detected within the first 4 days after infection occurs, which would reduce the severity of the disease, decrease economic losses, and increase animal welfare.

Although the algorithm with the best result (highest AUC) was Rule 1–30% of the Some2 strategy, which obtained a sensitivity of 95.2% and a specificity of 85.7%, depending on the needs of each farm, other algorithms with a higher specificity (up to 92.9%) could be used, maintaining good sensitivity (81%).

For its application at the farm level, the industry still needs to develop biosensors like those already existing for SCC and EC to be able to analyse this enzyme automatically.

## Figures and Tables

**Table 1:** Gland health status of ewes in the pre-experimental and experimental phase.

	Gland Health Status (GHS)	Ewes	Glands
Pre-experimental phase	Healthy	23 + 3 glands ^1^	49
	Unilateral infection	0	0
	Bilateral infection	0	0
Experimental phase (after subjecting glands to UHS)	Healthy	14	28
	Unilateral infection	3	3
	Bilateral infection	9	18
	Total infected	12	21

^1^ Sheep that had only one functional gland. UHS: unhealthy situation.

**Table 2:** Evolution of N-acetyl-β-D-glucosaminidase (μM/min per mL) and somatic cell count (×1000 cell/mL) (median (1st, 3rd quartiles)) according to gland health status and period relative to the onset of infection.

**NAGase**					
	**Days Prior to UHS**		**Days After UHS**		
**GHS**	**Previous 14–7 d**	**Previous 3 d**	**Following 4 d**	**Following 7–14 d**	**Following 21–28 d**
0-Healthy	45.55 (35.99, 62.14)	49.16 (36.86, 65.55)	51.57 (36.34, 67.31)	65.93 (48.56, 88.82)	74.06 (54.78, 97.06)
1-Healthy colInf	49.82 (35.39, 49.88)	39.13 (28.85, 48.36)	50.36 (22.12, 61.72)	61.35 (39.35, 76.42)	58.29 (49.83, 63.53)
2-Inf colhealthy	40.54 (29.79, 48.84)	61.46 (51.35, 73.88)	95.59 (51.09, 191.15)	76.49 (60.52, 93.51)	64.84 (47.91, 71.61)
3-InfBil	32.88 (23.4, 44.63)	27.44 (20.55, 35.86)	105.69 (60.19, 198.68)	54.47 (42.33, 79.74)	59.85 (41.71, 86.96)
**SCC (×10^3^ cell/mL)**					
	**Days prior to UHS**		**Days after UHS**		
**GHS**	**Previous 14–7 d**	**Previous 3 d**	**Following 4 d**	**Following 7–14 d**	**Following 21–28 d**
0-Healthy	54 (44.25, 77.5)	75 (56, 108)	115 (79, 187)	100 (67.5, 128.5)	90 (58.75, 121)
1-Healthy colInf	87 (75, 172)	97 (67, 120)	159.5 (82.75, 259)	101 (88.25, 242.75)	120 (87, 195)
2-Inf colhealthy	76 (66, 804)	367 (97, 1412)	1674 (147.5, 7107)	154.5 (128.5, 335.75)	107.5 (87, 131)
3-InfBil	45 (30.25, 63.75)	44 (37, 62)	2601 (697, 7624)	85.5 (68, 306.75)	79 (51.5, 457.25)

GHS: Glandular health status. 0-Healthy: Both collateral glands free of infection throughout the experiment. 1-Healthy colInf: Free of infection throughout the experiment whose collateral was infected during the experiment. 2-Inf colHealthy: Infected during the experiment, whose collateral remained free of infection. 3-InfBil: Both collateral glands infected during the experiment.

**Table 3:** Correlation between N-acetyl-β-D-glucosaminidase and SCC based on somatic cell values in sheep milk.

Bootstrapping Correlation Coefficient r, [95% Confidence Interval]	R	n
SCC ≤ 400 (×1000 cell/mL)	0.41 *** [0.34, 0.50]	506
SCC > 400 (×1000 cell/mL)	0.65 *** [0.56, 0.79]	102
Total	0.79 *** [0.76, 0.82]	608

Abbreviations: r: Correlation coefficient; n: Number of observations used; SCC = somatic cell count. *** *p* < 0.001.

## Data Availability

The original contributions presented in the study are included in the article, further inquiries can be directed to the corresponding author.
